# Sudden Death after Uterine Injection

**Published:** 1876-01

**Authors:** 


### MISCELLANEOUS.

SUDDEN DEATH AFTER UTERINE INJECTION OF IRON.—The following case was reported by Dr. Cederschiold before the Swedish Medical Society, and it is of interest, as being another instance where injection of fluids into the uterine cavity has been followed by sudden death. The patient was pregnant for the second time. A considerable hemorrhage followed the birth of the child, the uterus did not contract fully, and the fundus could be felt over the pubes. Ergot was of little use, and the hemorrhage recurred from time to time. Eighteen days later a strong solution of the perchloride of iron (1:7) was injected into the uterus. Every precaution was taken; the syringe was freed from air, the pressure on the piston was gradual, etc., but when the injection was half completed the woman suddenly complained of pain in the breast, stretched backward, drew a few short breaths, and was dead.

A post-mortem examination was made the next day. The small intestines were actively congested; a few spoonfuls of thin blackish fluid were found in the fossa of Douglas, and on the peritoneum in that vicinity there were numerous black spots. The uterus was pretty firmly contracted, was 11 centimetres long, 9 centimetres broad at the fundus, its greatest thickness 4 to 5 centimetres, the uterine walls at the middle 14 millimetres, at the fundus 10 mill-metres. The interior of the uterus and vagina was stained dark-brown. The interior of the uterus was uneven and covered with a reddish granulation tissue, with the exception of the sides and fundus, where three superficial oval ulcerated surfaces were found, each 4 to 5 centimeters long. Here the uterine substance was exposed, had a ragged surface, in the centre of which there were leaf-like, somewhat firm structures 1 centimetre high and 3 long. These were intimately united with the underlying tissues, and consisted of organic muscular fibres. At the sides of these formations there were open-mouthed vessels, some of them large enough to admit a fine sound, which then passed into the larger veins of the uterus. These were slit up and followed into the hypogastric and iliac veins, and the vena cava inferior. The blood in these veins was found markedly coagulated, and stained brown. Bubbles of air were also found in them. The same condition was found in the right side of the heart. The lungs and other organs presented nothing abnormal.—*Hygiea*, August, 1875.—*Med. Record.*

